# Comparative Metabologenomics Analysis of Polar Actinomycetes

**DOI:** 10.3390/md19020103

**Published:** 2021-02-10

**Authors:** Sylvia Soldatou, Grímur Hjörleifsson Eldjárn, Andrew Ramsay, Justin J. J. van der Hooft, Alison H. Hughes, Simon Rogers, Katherine R. Duncan

**Affiliations:** 1 Strathclyde Institute of Pharmacy and Biomedical Sciences, University of Strathclyde, Glasgow G4 0RE, UK; s.soldatou@rgu.ac.uk (S.S.); a.hughes@strath.ac.uk (A.H.H.); 2 School of Computing Science, University of Glasgow, Glasgow G12 8RZ, UK; 2332462H@student.gla.ac.uk (G.H.E.); andrew.ramsay@glasgow.ac.uk (A.R.); Simon.Rogers@glasgow.ac.uk (S.R.); 3 Bioinformatics Group, Wageningen University, 6708 PB Wageningen, The Netherlands; justin.vanderhooft@wur.nl

**Keywords:** Actinobacteria, marine, Polar, genomics, metabolomics, specialised metabolites

## Abstract

Biosynthetic and chemical datasets are the two major pillars for microbial drug discovery in the *omics* era. Despite the advancement of analysis tools and platforms for multi-strain metabolomics and genomics, linking these information sources remains a considerable bottleneck in strain prioritisation and natural product discovery. In this study, molecular networking of the 100 metabolite extracts derived from applying the OSMAC approach to 25 Polar bacterial strains, showed growth media specificity and potential chemical novelty was suggested. Moreover, the metabolite extracts were screened for antibacterial activity and promising selective bioactivity against drug-persistent pathogens such as *Klebsiella pneumoniae* and *Acinetobacter baumannii* was observed. Genome sequencing data were combined with metabolomics experiments in the recently developed computational approach, NPLinker, which was used to link BGC and molecular features to prioritise strains for further investigation based on biosynthetic and chemical information. Herein, we putatively identified the known metabolites ectoine and chrloramphenicol which, through NPLinker, were linked to their associated BGCs. The metabologenomics approach followed in this study can potentially be applied to any large microbial datasets for accelerating the discovery of new (bioactive) specialised metabolites.

## 1. Introduction

More than 80 years after the first reported case of sulphonamide-resistant bacterial strains [1], antibiotic resistance is now a global threat to human health, projected to cause 10 million deaths annually by 2050 [2,3]. Historically, microorganisms have been a source of over 22,000 biologically active metabolites, including antibiotics, isolated from terrestrial and marine strains [4]. In particular, the order Actinomycetales (actinomycetes) has been shown to produce structurally diverse specialised metabolites which exhibit a wide range of biological activities. Indeed, more than 7000 metabolites have been isolated from the genus *Streptomyces* and approximately 3000 metabolites from the “rare” (due to their lower isolation frequency) actinomycete genera [5]. Actinomycetes have an average genome size of over 5 Mb. However, this number varies greatly between genomes which can reach up to 12 Mb in some *Streptomyces* species [6]. Actinomycetes dedicate 0.8–3.0 Mb of their whole genome to specialised metabolite production, which has been shown to result in 20–50 Biosynthetic Gene Clusters (BGCs) per strain [7]. A recent study of 21 rare marine actinomycetes isolated from temperate and sub-tropic marine environments unveiled diverse and numerous BGCs, with *Actinomadura* spp. and *Nocardia* spp. containing 44 and 38 BGCs per strain, respectively. Interestingly, only three percent of the BGC families overlapped between the *Streptomyces* strains and the rare actinomycetes, suggesting an exciting resource for biosynthetic novelty [8]. Moreover, analysis of 119 genomes of the rare actinomycete genus *Salinispora* derived from various sub-tropic and tropic locations revealed 176 BGCs, of which only 24 were linked to their respective products [9], indicating a potential resource for novel metabolites.

Although actinomycetes from the (sub-)tropics have been extensively studied and shown to be a promising source of biological and chemical novelty, Actinobacteria isolated from Polar regions also show potential for affording new biologically active specialised metabolites. Actinobacteria accounted for 5% of the total microbial community present in Antarctic sediment samples as revealed by high-throughput 16S rRNA gene sequencing, with the *Salinibacterium* genus being amongst the most abundant [10]. In terms of isolating rare actinomycetes from Polar habitats, a study of ancient Antarctic and sub-Arctic sediment samples yielded 50 bacterial strains, of which 39 belonged to rare actinomycetes genera (*Microbacterium*, *Dietzia*, *Rhodococcus*, and *Pseudonocardia*) [11]. Regarding the chemical potential, molecular networking indicated rare actinomycetes from sub-Arctic and Antarctic sediments to be a rich source of metabolites [11,12]. Since 2001, a total of twenty-nine new metabolites have been isolated from Antarctic and sub-Arctic bacteria, with 13 being produced by marine actinomycetes [13]. For example, the rare actinomycete *Nocardia dassonvillei* BM-17 isolated from an Arctic sediment sample yielded a new phenazine derivative with significant antifungal and cytotoxic activity [14]. Two new α-pyrones were isolated from an Antarctic *Nocardiopsis* strain [15], whereas the seaweed-derived *Nocardiopsis* sp. 03N67 collected in the Arctic Ocean afforded a new diketopiperazine, cyclo-(L-Pro-L-Met), which showed promising anti-angiogenesis activity [16].

It is widely known that variations in cultivation parameters can induce the expression of so-called ‘silent’ BGCs, for which the biosynthetic enzymes have been identified but no natural product has been isolated from laboratory cultures [17]. Hence, changes in abiotic factors such as nutrient availability (carbon, nitrogen, trace-elements), temperature, salinity, and pH have been shown to influence the production of such ‘cryptic’ specialised metabolites. Moreover, the chemical profile of a microorganism can depend on the culture vessels, shaking conditions, and aeration, as well as on co-culturing techniques [18]. For example, when glucose was substituted for glycerol in the ISP2 growth medium, the liquid culture of *Streptomyces* sp. C34 isolated from the Atacama Desert, yielded ansamycin-type polyketides [19]. Furthermore, the marine-derived *Streptomyces* sp. CHQ-64 was found to produce new biologically active polyene-polyols and hybrid isoprenoid alkaloids when cultured under shaking, whereas static fermentation yielded only one new metabolite [20,21]. Therefore, the “One Strain Many Compounds” (OSMAC) approach [22] has been a successful addition in the microbial drug discovery pipeline

Mass spectrometry approaches are often used to compare multi-strain metabolomics. Molecular networking using the Global Natural Products Social Molecular Networking (GNPS) infrastructure [23] has been deemed a valuable tool in the discovery of new metabolites [24] as it provides rapid dereplication [25] and identification of unknown parent ions. Clarinoside, a new pentalogin from the plant *Mitracarpus scaber* Zucc [26], retimycin A, a non-ribosomal peptide from *Salinispora arenicola* [27], and deoxyphorbol ester derivatives from *Euphorbia dendroides* [28] are a few of the specialised metabolites discovered using MS-guided isolation based on the GNPS platform. Recently, further analysis tools have been implemented on the GNPS infrastructure, such as MS2LDA which provides fragmentation patterns of commonly co-occurring mass fragment peaks and/or neutral losses that often represent molecular substructures (Mass2Motifs) [29], and Network Annotation Propagation (NAP) [30] which improves in silico fragmentation of the input data. The MolNetEnhancer workflow was introduced to combine the outputs of the above-mentioned tools and add (putative) chemical class annotations to molecular families in the molecular network [31]. 

The sequencing of the first *Streptomyces* genome in 2002 [32] paved the way for the discovery of further microbial natural products based on genomic data. The continuous development of genome sequencing technology has led to a wealth of genomic data which has motivated the development of sophisticated mining tools that can augment the search and discovery of novel specialised metabolites [33,34]. Several of these [35] are publicly accessible, enabling thorough and targeted genome mining of complex bacterial genomes, including antiSMASH for the identification of secondary metabolites BGCs [36], ARTS for high-throughput screening of bacterial genomes in reference to antibiotic production [37], and BiG-SCAPE for clustering BGCs into Gene Cluster Families (GCFs) [38]. 

The linking of genome and metabolome mining outcomes to accelerate natural products discovery has shown great promise over the last decade, with several tools contributing to bridging the gap between BGCs and mass spectra [39]. Although the term “metabologenomics” was officially introduced in 2016 to describe correlations between BGCs and metabolites [40], research on this approach started earlier. A study of actinomycete strains in which their genomic data were linked with MS profiles, led to the identification of GCFs for the previously reported natural products desertomycins and oasamycins, for which the corresponding BGCs were unknown [41]. The authors were also able to isolate and characterise a new chlorinated metabolite, tambromycin, and correlate it with its BGC in 11 actinomycete strains using metabologenomics [40]. Another successful study used a combination of molecular networking and pattern-based genome mining approach from which arenicolide A was linked to an uncharacterised BGC (PKS28) and the new metabolite, retimycin A was identified, characterised and linked to the known NRPS40 pathway [27].

Herein, we introduce a novel unsupervised -*omics* integration method to link tandem mass spectrometry data to BGCs to accelerate the analysis of large microbial natural products datasets. NPlinker, a newly introduced software framework [42] was applied for the first time to bridge the large metabolomics and genomics datasets of marine Polar Actinobacteria. With the aid of the novel approach Rosetta, links between spectra and BGCs for chloramphenicol and ectoine were established. Molecular networking of the 100 metabolite extracts derived from applying the OSMAC approach, showed growth media specificity and potential chemical novelty was suggested. Moreover, the metabolite extracts were screened for antibacterial activity and promising selective bioactivity against drug-persistent pathogens such as *Klebsiella pneumoniae* and *Acinetobacter baumannii* was observed.

## 2. Results

### 2.1. Phylogenetic Analysis

**Figure 1:**
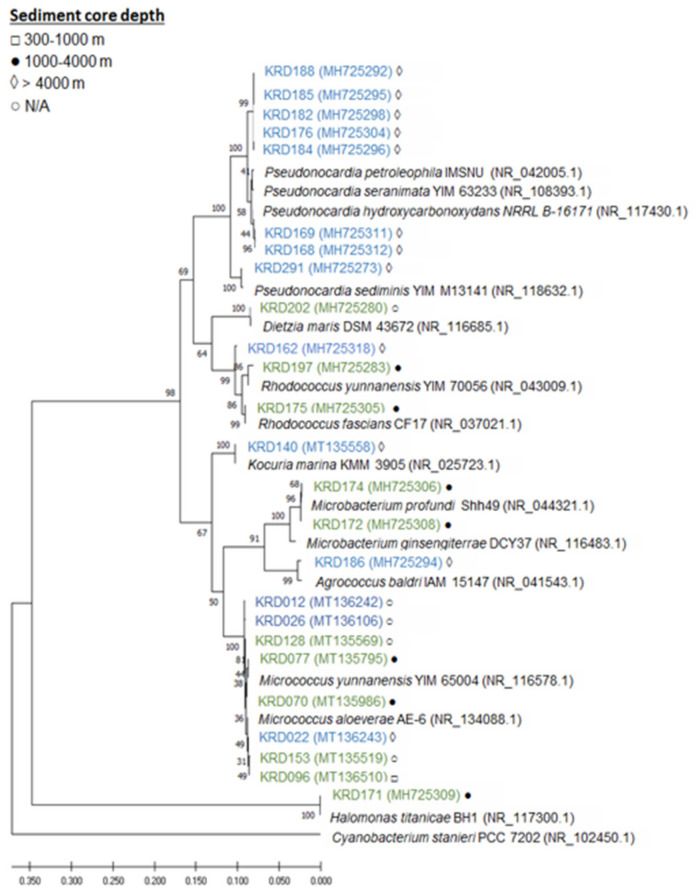
Maximum likelihood tree based on 16S rRNA gene sequences of 25 strains isolated from Antarctic (blue) and Arctic (green) sediment samples. Strain numbers are followed by a symbol indicating the depth at which the sediment samples were collected from: □ 300–1000 m, ● 1000–4000 m, ◊ > 4000 m and ○ N/A (information missing). The accession number is shown in brackets following the strain name.

Twenty-five strains, 14 Antarctic and 11 Arctic, were selected from a larger collection of Polar marine sediment bacteria mainly consisting of Actinobacteria [11], based on taxonomy, isolation location (depths ranging from 388 to 4730 m) and previously known metabolic profile of the strains. The selected strains belonged to seven rare actinomycete genera—*Pseudonocardia* (eight strains), *Micrococcus* (seven strains), *Rhodococcus* (three strains), *Microbacterium* (three strains), *Kocuria* (one strain), *Agrococcus* (one strain), *Dietzia* (one strain). This genus-level delineation was well supported with bootstrap values over 67% and all strains clade with their respective reference sequences. Additionally, one strain of the phylum Proteobacteria, belonging to the *Halomonas* genus, was included in the study (Figure 1, Table S1). In terms of core depth (Figure 1), the two *Agrococcus*, one *Kocuria* and the eight *Pseudonocardia* strains were only isolated from the deepest sediment cores at a depth greater than 4000 m, and the one *Halomonas* and the two *Microbacterium* strains were only isolated from core depths of 1000–4000 m, while no pattern was observed for the genera *Micrococcus* and *Rhodococcus*. Although these observations are interesting, larger strain numbers would be required to draw statistical conclusions.

### 2.2. Genome Mining

Genome assembly was carried out using SPAdes and due to the large numbers of contigs obtained, MeDuSa was utilised for genome scaffolding, using reference strains with >95% similarity based on 16S rRNA sequencing data (Table S2). No reference strains with >95% sequence similarity could be identified for the *Pseudonocardia* strains; therefore, they were eliminated from genome mining to avoid possible discrepancies.

**Figure 2:**
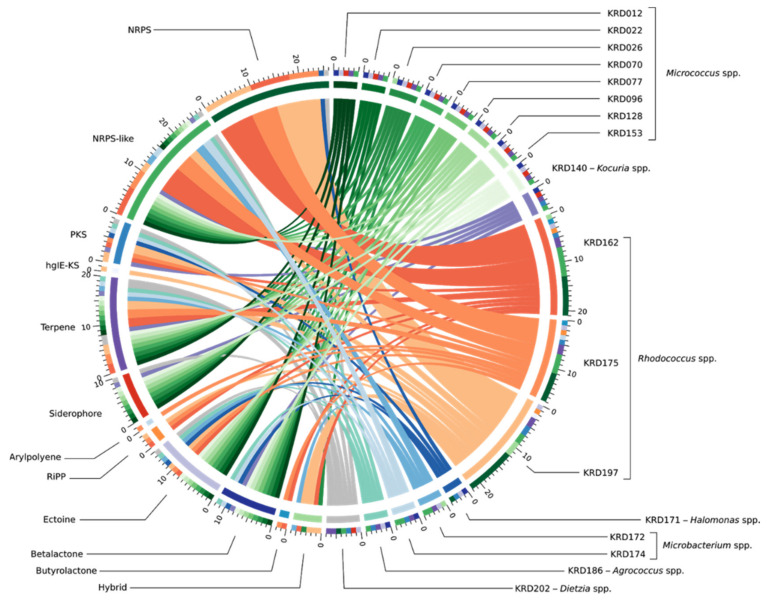
BGC diversity by NP class and strain taxonomy across 17 Polar strains. The band colour depicts taxonomy at the genus-level *Micrococcus* spp. (green), *Kocuria* sp. (purple), *Rhodococcus* spp. (orange), *Halomonas* sp. (dark blue), *Microbacterium* spp. (light blue), *Agrococcus* sp. (teal) and *Dietzia* sp. (grey). Each coloured band can be traced from the organism (right half of the circle) to the types of BGCs found in that genome (left half of the circle). The width of the band represents the number of BGCs of that NP class. The outer rings on the left of the diagram show the number of the BGC types found in each microbial genome. BGCs are colour coded based on the NP classification.

Genome mining of the seventeen Polar rare Actinobacteria and the Proteobacteria (*Pseudonocardia* strains excluded) revealed a total of 133 BGCs including NRPS, PKS, terpene and RiPP classes. Interestingly, 37% of the total BGCs showed no homology to any BGC within the MiBIG database and a further 30% suggested homology ranging from 2% to 63% to known antibiotics. The biosynthetic diversity per strain is shown in the Circos diagram (Figure 2). The width of the bands indicates the number of BGCs within each Natural Product (NP) class which, as expected, is positively correlated to genome size. The lowest number of BGCs was observed for small genomes such as the *Micrococcus* (2.4–2.7 Mbp) and *Agrococcus* (3.1 Mbp) both of which had five BGCs, whereas the three *Rhodococcus* strains (5.4–6.7 Mbp) revealed the largest number (17–22) of BGCs. Moreover, strains belonging to the same genus showed BGCs of the same NP class (Table S3). The ectoine pathway was observed in almost all genomes except the *Kocuria* (KRD140) and the *Microbacterium* (KRD174) strains. A similar pattern was observed for the terpene BGC which was present in the genome of all Polar isolates except the *Halomonas* strain (KRD171) (Table S3). The most abundant NP class was NRPSs which were not evenly distributed among all strains, as the smaller genomes such as *Micrococcus* and *Microbacterium* did not show any NRPS BGCs, although at least one NRPS-like fragment was identified in their genomes. On the other hand, larger genomes such as the *Rhodococcus* strains revealed a high number of NRPS BGCs (up to 10). Identification of siderophores based on bioinformatic analysis can often be challenging as many siderophores are produced through NRPS pathways, thus antiSMASH identifies them as NRPS and not siderophores [8,43]. Indeed, antiSMASH identified two NRPS clusters in one *Halomonas* strain (KRD 171) and one *Rhodococcus* strain (KRD 197) that have 53% and 63% gene homology to the serobactin and heterobactin siderophore pathways, respectively. Only 10 BGCs belonging to the PKS family were observed, of these, five were identified as Type I PKS, one Type II PKS, three Type III PKS, and one heterocyst glycolipid synthase-like PKS (hgIE-KS).

**Figure 3:**
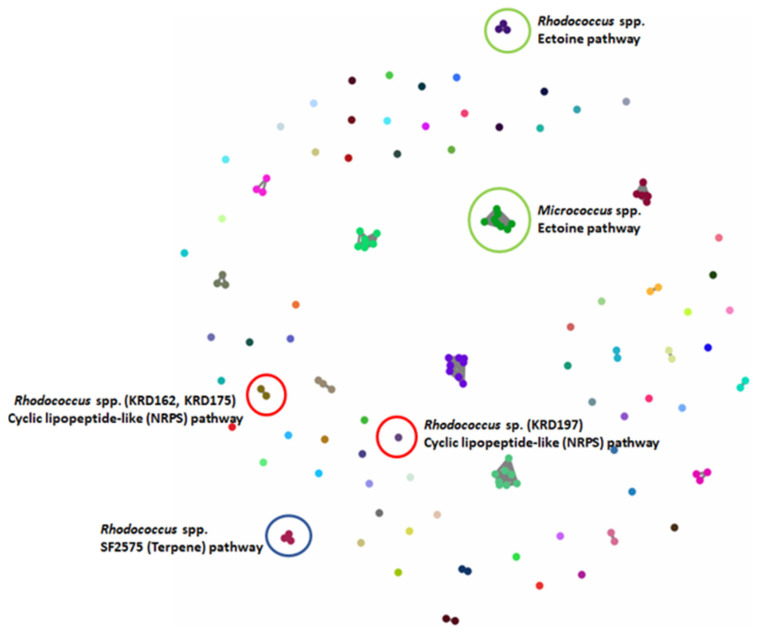
BiG-SCAPE analysis of 17 strains. The 133 BGCs; 53 NRPS/NRPS-like, 20 Terpene, 10 PKS, 3 RiPP and 47 others (including BGCs such as ectoine, siderophore, betalactone) were clustered in 80 GCFs. Examples of GCFs of interest are highlighted in coloured circles; red represents the *Rhodococcus* spp. NRPS BGC corresponding to a potentially new cyclic lipopeptide, green represents the ectoine pathway found in all strains (here highlighted for the *Micrococcus* and *Microbacterium* spp.) and blue the terpene BGC found in all three *Rhodococcus* strains with low homology to the known antibiotic SF2575.

The BGCs of the 17 Polar strains were further analysed using BiG-SCAPE, which resulted in 80 GCFs, with 46% shown as singletons. As expected, there were BGCs present in strains belonging to the same genus, that clustered in the same GCF. For example, the ectoine BGCs present in the eight Micrococcus strains clustered in one GCF and the ectoine BGCs of the three *Rhodococcus* strains were represented as an additional GCF (green circles in Figure 3). The three *Rhodococcus* strains had a terpene BGC which showed low homology (6%) to SF2575 BGC from the soil-derived *Streptomyces* sp. SF2575 [44] (blue circle in Figure 3). Although the homology with the known biosynthetic pathway is low, the fact that it is shared by all three BGCs from the different *Rhodococcus* strains, implies that the strains may produce the same or similar metabolite(s) to the tetracycline antibiotic SF2575 [45]. Additionally, the three *Rhodococcus* strains (KRD12, KRD175, KRD197) showed an NRPS BGC that shows low homology (11%) to the chloramphenicol BGC from *Streptomyces venezuelae* ATCC 10712 [46]. Interestingly, only the gene clusters of KRD175 and KRD162 were grouped in the same family, whereas the corresponding BGC of KRD197 was shown as a singleton (red circles in Figure 3) even running BiG-SCAPE with a high cut off (0.7). Further investigation of the antiSMASH data showed that the predicted metabolites for the NRPS genes of interest could be cyclic lipopeptides, which often exhibit antibiotic properties [47].

### 2.3. Antibacterial Activity and Parent Ion Distribution

**Figure 4:**
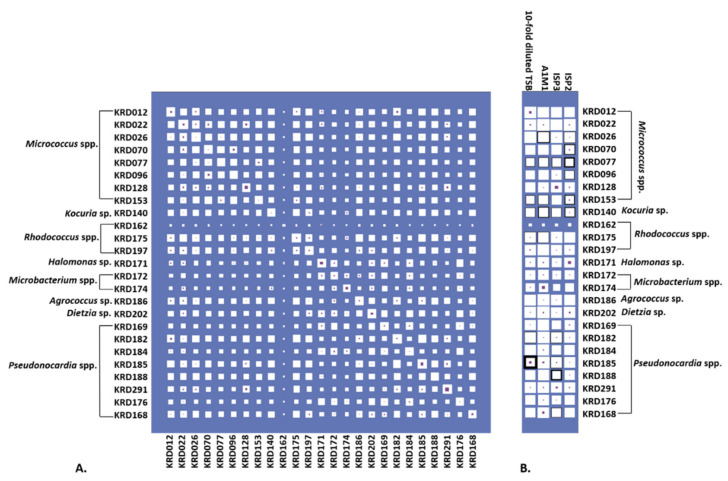
(**A**) Hinton diagram showing the number of parent ions (proportional to the size of white box) produced by each strain (max. 1041, min. 543 parent ions) and shared by each pair of strains (max. 670, min. 104 parent ions. Number of parent ions specific to that strain (or pair) are proportional to the size of the inner purple box. (**B**) Hinton diagram showing the number of parent ions by strain across each media (white box). Parent ions specific to only that strain-medium are also shown (purple box). The thickness of black box outline corresponds to the number of ESKAPE pathogens bioactivity was observed against (ranging from 1 to 6). For example, the bacterial metabolite extract for KRD185 in diluted TSB was found to be bioactive against all six pathogens, but no bioactivity was observed when the same strain was cultured in any of the other tested media.

Culturing all strains in four growth media resulted in 100 metabolite extracts, of which 72% exhibited activity against six pathogenic bacteria (*Escherichia coli*, *Staphylococcus aureus*, *Klebsiella pneumoniae*, *Acinetobacter baumanii*, *Pseudomonas aeruginosa* and *Enterococcus faecalis*) known as the “ESKAPE” pathogens [48]. Specifically, 39% of the biologically active strains (28% of the total number) showed antibacterial activity against only one pathogen. The same percentage of active extracts inhibited the growth of one or more pathogens under only one cultivation condition. The inhibition zones ranged from 0.1 to 2.1 cm (Table S4). Most of the bacterial metabolite extracts were active against *S. aureus* with the *Pseudonocardia* strain KRD185 showing the largest inhibition zone (2.1 cm). Moreover, the 10-fold diluted TSB extract of strain KRD185 showed promising antibacterial activity against *K. pneumoniae* (0.9 cm) and *A. baumannii* (1.5 cm), whereas the A1M1 and 10-fold diluted TSB extracts of *Rhodococcus* strain KRD175 were selectively active against *K. pneumoniae* (Table S4). The occurrence of parent ions in relation to the bioactive extract is shown in Figure 4 where Hinton diagrams are illustrating the number of parent ions produced only by each strain (strain specific), as well as shared between two strains (Figure 4A). Strains belonging to the same genus shared the highest number of produced parent ions, which of course varies between the genera. For example, within the *Micrococcus* genus, strains KRD022 and KRD026 share the highest number of parent ions (649 in total), whereas strains KRD128 and KRD096 share only 287 parent ions. For the *Pseudonocardia* spp. isolates, there is a wide variation in the number of shared parent ions (163–632), where strain KRD176 shares 632 ions with KRD196 and only 163 with KRD291. Variations ranging from 104 to 649 shared parent ions also occurred between strains of different genera, as expected. The *Microbacterium* sp. strain KRD174 shared 104 and 123 parent ions with *Micrococcus* sp. KRD096 and *Pseudonocardia* sp. KRD291, respectively, representing the lowest number of shared ions. On the other hand, the *Rhodococcus* strain KRD175 showed the largest number of shared parent ions (649) with strain KRD022 of the *Micrococcus* genus. The sole *Kocuria* sp. isolate, KRD140, shared only 199 parent ions with *Pseudonocardia* strain KRD176, but shared more than 400 ions with three *Pseudonocardia* strains (KRD182, KRD185, KRD188). Amongst the *Pseudonocardi*a strains, KRD182 showed the highest number of shared parent ions with *Micrococcus* spp. (356–607 parent ions), and *Rhodococcus* strains (465–612 parent ions). The Hinton diagram in Figure 4B demonstrates the number of parent ions produced by strains under each growth condition (white box), as well as the number of specific parent ions per strain grown under each growth condition (purple box) and the observed bioactivity (black outline). The 10-fold diluted TSB metabolite extract of KRD185 (*Pseudonocardia* sp.) had the highest bioactivity, with a zone of inhibition of 2.1 cm, and it produced the highest number of total (803) and unique (29) parent ions in that growth condition when compared to the rest of the studied isolates. Although the *Micrococcus* strain KRD022 produced the highest numbers of parent ions in ISP2 (819) and A1M1 (864) media, it did not exhibit any biological activity against the pathogenic bacteria. These two examples indicate that bioactivity is not necessarily related to the highest number of produced metabolites (parent ions). 

### 2.4. Molecular Networking

**Figure 5:**
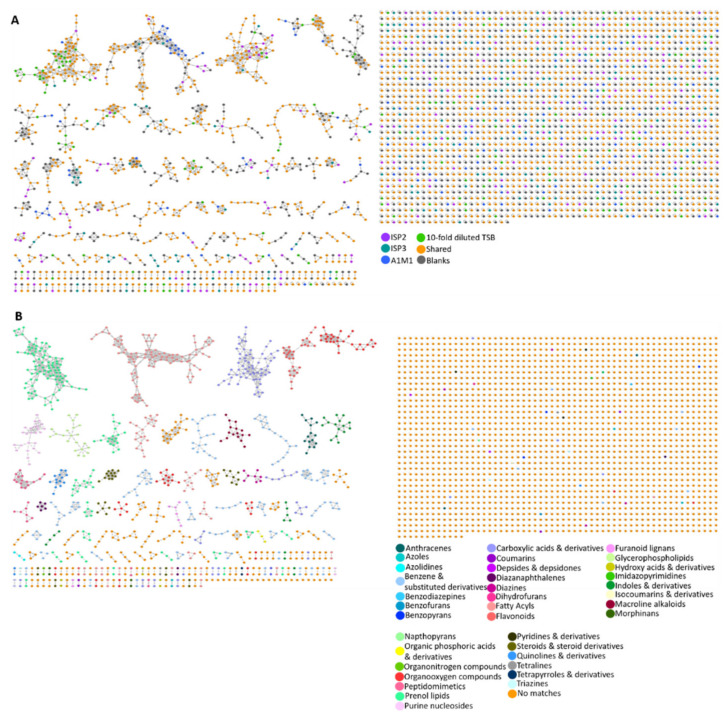
(**A**) Molecular network of 3107 parent ions produced by 25 strains cultured in four media and those found in media and solvent blanks. Nodes are colour coded based on media: ISP2, ISP3, A1M1 and 10-fold diluted TSB. Grey nodes represent media components, whereas orange nodes represent parent ions that are found in more than one different medium. (**B**) Nodes are colour coded based on 36 chemical class terms annotated using MolNetEnhancer workflow. Orange nodes represent parent ions that had no matches with any chemical class.

A molecular network of all 100 microbial metabolite extracts (25 strains cultured in four media), in addition to the media and solvent blanks, consisted of 3107 parent ions (nodes). There were 721 nodes that were excluded from the data analysis as they corresponded to parent ions present in the media and solvent blanks. Of the total number of parent ions produced (i.e., not present in the blanks, 2386), 414 of these nodes were singletons indicating that their fragmentation pattern did not correlate with that of any other parent ion, suggesting chemical novelty within the dataset. After ions in the media controls were excluded, 23% (549) of ions were produced by strains grown in all four media. A further 65% (1551) of ions were produced in more than one medium (i.e., not media specific). Interestingly, the percentage of nodes which were media-specific was almost constant across ISP2 (8.3%), ISP3 (8.8%), and 10-fold diluted TSB (8.0%) media, whereas 6.3% of the produced ions were present in the metabolite extracts derived from A1M1 medium. The MolNetEnhancer workflow showed 38 (putative) chemical classes annotated in the molecular network (Figure 5B and Figure S2). Almost 71% of the produced ions did not match any chemical classification which implies chemical novelty and is in accordance with the low number of library hits generated by the GNPS molecular network. Fatty acyls, and benzene and substituted derivatives represented 4% and 5% of the produced ions, whereas prenol lipids covered 6% of the chemical classes identified in the network. 

### 2.5. Computational Pattern Matching

**Figure 6:**
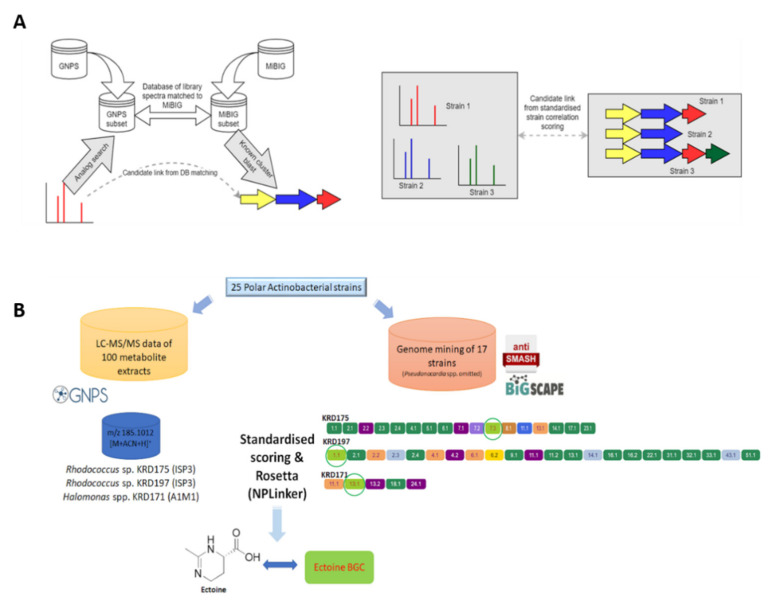
(**A**) Molecular families (MFs) are created through molecular networking using the GNPS infrastructure and parent ions of interest are identified through dereplication using GNPS embedded libraries and the Rosetta tool. Genome mining data (antiSMASH) of the Polar strains were clustered in GCFs using BiG-SCAPE. The GNPS (MFs) and BiG-SCAPE (GCFs) outputs are then analysed with NPLinker to rank potential MF-GCF links using two scoring functions (standardised strain correlation linking and Rosetta scoring). (**B**) The ectoine metabolite produced by two *Rhodococcus* spp. (KRD175 and KRD197) when cultured in ISP3 and one *Halomonas* sp. (KRD171) cultured in A1M1 was linked with its corresponding ectoine BGC via NPLinker.

A recently introduced software framework, NPLinker, was utilised to suggest links between spectra of interest and their corresponding GCFs and therefore BGCs [42]. Initially, analysis was carried out using the standardised strain correlation scoring method which yielded potential MF-GCF links based upon correlating strain presence and absence. This approach greatly narrowed the space of links requiring investigation. Further analysis of the suggested links based on biosynthetic knowledge allowed the BGCs to be identified that were likely to be most relevant to the metabolite of interest. Specifically, the GNPS infrastructure allows parent ion clustering into molecular families and comparison of observed spectra with GNPS embedded libraries. Simultaneously, BGCs were clustered into GCFs via BiG-SCAPE. The generated MFs and GCFs were then uploaded to NPLinker where potential MF-GCF links were ranked based on two scoring functions; standardised strain correlation scoring and the here introduced, novel approach named Rosetta scoring (Figure 6A).

#### 2.5.1. Computational Pattern Matching Using the Standardised Strain Correlation Scoring Method

The parent ion (*m*/*z* 547.3815) produced by *Microbacterium* sp. KRD174 cultured in A1M1 showed spectral similarity to the GNPS spectrum CCMSLIB00000569369 suggesting it was an antimycin-related metabolite. Through NPLinker, it was shown that this metabolite could potentially be linked with the NRPS-like, betalactone, t3PKS and terpene BGCs (KRD174). Although the standardised strain correlation score for all links was high (2.7–4, with 4 being the maximum value observed in the dataset), when this information was combined with the fact that antimycins are produced by an NRPS/PKS hybrid [49], it was hypothesised that the betalactone and terpene BGCs were less likely to be involved in the biosynthesis of the metabolite of interest. Of course, further validation studies are required to confirm the responsible BGC. Similarly, another metabolite (*m*/*z* 521.3294) showed similarity with GNPS spectrum CCMSLIB00004710288 for conglobatin (MIBiG ID: BGC0001215), suggested that it could potentially be structurally related to the known macrolide conglobatin originally isolated from the antibiotic-producing *Streptomyces conglobatus* [50]. The metabolite of interest was produced by two *Micrococcus* strains, KRR022 and KRD026 (diluted TSB medium), in addition to *Rhodococcus* sp. KRD175 (diluted TSB medium) and two *Pseudonocardia* strains KRD184 and KRD291 (ISP2 medium). Using the standardised strain correlation scoring method, the spectrum was potentially linked with 14 GCFs; two from *Micrococcus* sp. (KRD026) and 12 from *Rhodococcus* sp. (KRD175). However, the highest standardised strain correlation linking score (2.1) was observed for the hybrid BGC arylpolyene-NRPS (KRD026) as well as for the NRPS, NRPS-like, arylpolyene and butyrolactone BGCs (KRD175). Considering that conglobatin biosynthesis is governed by an NRPS/PKS BGC [51], the arylpolyene-NRPS BGCs are most likely to be involved in the biosynthesis, but further studies would be required to validate this. These examples demonstrate that using spectral library matches with the standardised scoring method included within NPLinker can narrow down possible MF-GCF links and thus enable a more focused downstream analysis.

#### 2.5.2. Computational Pattern Matching Using Standardised Strain Correlation Scoring and the Rosetta Method

To further investigate the potential links between the genomics and metabolomics datasets of the Polar strains, an additional filter layer was added into NPLinker which allowed the use of the standardised strain correlation scoring method and the Rosetta hit list simultaneously. This approach led to linking spectrum ID 219769 (*m*/*z* 185.1012), putatively identified as ectoine ([M + CAN + H]^+^ adduct), via Rosetta, with the ectoine BGC in two *Rhodococcus* sp. (KRD175, KRD197) and Halomonas sp. (KRD171) strains. Interestingly, when using only the standardised scoring the same spectrum was linked to 40 GCFs. However, applying the additional Rosetta scoring method narrowed it down to two GCFs (Figure 6B). Moreover, Rosetta identified that spectrum ID 111427 (*m*/*z* 380.2794) could be structurally related to the known antibiotic chloramphenicol, originally isolated from *Streptomyces venezuelae* [52]. The parent ion of interest was present in the metabolite extracts of *Rhodococcus* sp. KRD175 and *Micrococcus* sp. KRD128 and was linked with the NRPS BGC (KRD175) which showed homology to the chloramphenicol BGC. It is important to note that the Rosetta scoring approach is limited by the number of MiBIG BGCs for which experimental spectra are available. Due to the relatively low number of publicly available spectra of microbial metabolites [53], the combined filtering approach (standardised score and Rosetta) could only identify links for ectoine and chloramphenicol to their corresponding BGCs. It must be pointed out that the Rosetta hits were a result of matching single MS fragments to publicly available MS/MS datasets (Table S5), hence the aforementioned metabolites could be only putatively identified. However, this workflow clearly shows the promise of the implemented method for analysing large genomics and metabolomics datasets.

## 3. Discussion

Over the years, it has been shown that the Arctic and Antarctic marine environment host a vast variety of Actinobacteria with great potential for producing novel chemistry with a wide range of biological activities [11,12,13]. Bioprospecting for new specialised metabolites from Polar strains has greatly improved by the advancement of publicly available tools for untargeted metabolomics [23] and genome mining [54], which are continuously under development to meet the rapidly evolving field of microbial natural products discovery. One of the main challenges of genome mining is the quality of the genome assembly and annotation which can affect the outcome of the analysis [55,56]. A large number of contigs in the genome assembly can lead to BGCs, especially PKS-I and NRPS, to be broken across pieces and not being identified by available software and tools. A great example of such issue was demonstrated by Baltz who showed that draft genomes containing large NRPS/PKS-I genes were incorrectly assembled due to being largely fragmented which resulted in overestimation of such BGCs by antiSMASH 3.0 [57]. However, since then, new updated versions of antiSMASH have been released in which the location of the gene cluster close to the contig edge is flagged. Moreover, the need for closed genomes is of paramount importance for accurate and reliable genome mining. However, long-read technologies are often required to achieve this, which comes with greater expense and their own drawbacks such as high error frequencies and reliability [58]. A recent study of nine Actinobacterial species, including three *Pseudonocardia* strains used short-read (Illumina MiSeq) and long-read (Oxford Nanopore MinION) sequencing technologies to analyse BGC fragmentation. The authors found that the MinION-based genome assemblies increased the sensitivity related to BGC annotation and reduced the number of fragmented BGCs. [56]. In this present study we omitted the *Pseudonocardia* strains from the genomic analysis due to lack of reference strains for genome scaffolding. Genome mining of the 17 non-*Pseudonocardia* strains revealed a wide diversity of BGCs with most of them having low homology to known BGCs which suggests biosynthetic and chemical novelty. Terpene BGCs were present in almost every genome, which was not surprising as recent studies have revealed a wide distribution of terpene synthases in bacteria which has led to the development of a new hidden Markov model for terpene synthases identification in bacterial genomes [58,59]. As expected, the number and variety of BGCs increased for larger genome sizes such as the *Rhodococcus* strains. However, it was unexpected to notice that smaller genomes such as *Micrococcus*, *Halomonas* and *Kocuria* were lacking PKS and NRPS BGCs as actinomycetes are known to produce metabolites encoded by those pathways [60,61]. A similar observation was made by Schorn et al. when studying rare marine actinomycetes [8]. Although small genomes might not look as promising from a natural products discovery perspective, it does not necessarily mean that they are not worth further investigation. The sponge-associated *Micrococcus* sp. was reported to produce a new antibacterial xanthone named microluside A [62] and marine *Halomonas* strains have yielded new antibacterial and cytotoxic metabolites named loihichelins A−F and aminophenoxazinones, respectively [63,64].

To further explore and investigate the observed BGCs in our Polar strains, analysis showed the ectoine BGC present in all genomes; this is known to be ubiquitous as the metabolite aids survival under extreme osmotic stress [65]. Moreover, the terpene BGC with high homology (66%) to a known carotenoid BGC was present in all *Micrococcus* strains and clustered in the same GCF. Carotenoids are terpenoids produced by all photosynthetic organisms and some non-phototrophic organisms, and have several applications as food colorants, feed supplements, nutraceuticals, and pharmaceuticals [66]. Terpene BGCs with homology (>37%) to the isorenieratene BGC were observed in the *Rhodococcus* strains and were clustered in the same GCF. Actinobacteria, and particularly *Streptomyces* spp., often bear isorenieratene BGCs in their genome that are usually silent, and there have been only a few cases in which these BGCs have been activated [67,68]. Furthermore, the genomic data of the three strains belonging to the genus *Rhodococcus* suggest the presence of NRPS BGCs which could potentially encode for cyclic lipopeptides. Such metabolites are of great importance in drug discovery with the example of daptomycin, originally isolated from the soil-derived *Streptomyces roseosporus* [69], which has been approved by the FDA as an antibacterial agent against Gram positive pathogens [70].

For over 30% of the BGCs within our dataset, the most similar known cluster encoded for an antibiotic. Of this, almost half showed low homology (<10%) with known BGCs. This is an exciting finding suggesting that the rare actinomycete strains derived from Polar marine sediments can potentially be a fruitful source of novel chemistry. It is worth noting that extracting metabolites from culture broth in organic solvents was proven to be a more effective and reliable method to assess biological activity (disc diffusion assay) than an agar plug assay [71]. Although genome mining of the *Rhodococcus* spp. showed promising potential for producing metabolites, the bioassay data did not fully support this. As only strain KRD175 exhibited moderate but selective activity against *K. pneumoniae*. This could be because the BGCs encoding for antibiotics remained silent or the biologically active compounds were produced in low amounts that were not sufficient to inhibit the growth of the pathogens. Moreover, the bacterial metabolite extracts mostly inhibited the growth of *S. aureus*, whereas only a few showed inhibitory effects against *K. pneumoniae* and *A. baumannii*, which are two of the most drug-persistent pathogenic bacteria [72,73]. To the best of our knowledge, there are only a few published reports on the inhibitory effects of microbial specialised metabolites on *A. baumannii* [74,75,76] but none on *K. pneumoniae*; and therefore, the Polar strains with such activity show promise to combat these pathogens. 

Linking genomic and metabolomics datasets of actinomycete strains for specialised metabolite discovery has been introduced only recently [41]. However, there is increased interest in the scientific community to further explore this niche research field by generating automated methods for correlating these complex datasets and ranking promising MF-GCF links for further investigation. Targeted linking and automated approaches for accelerating drug discovery have been reviewed [39,53]. Recently, metabolomic and genomic data of 72 isolates belonging to the rare actinomycete genus *Planomonospora* were analysed using publicly available tools to link specialised metabolites to their corresponding BGCs [77]. The authors were able to manually pair siomycin congeners to a RiPP BGC and a new salinichelin-like metabolite to the known BGC encoding for erythrochelin. In the present study, the newly developed software, NPLinker, was used to link our experimental datasets and prioritise strains for further chemical and biosynthetic investigation. The filtering approaches that were implemented (standardised strain correlation score and Rosetta) established links for ectoine and chloramphenicol to their corresponding BGCs but were not yet sufficient to link the potentially new identified metabolites (antimycins-like and conglobatin-like compounds) to GCFs as publicly available spectra of microbial metabolites are almost non-existent and remain mostly hidden in supplemental figures in literature. Van Santen et al. [78], among others, discussed the need for data sharing within the scientific community which will allow the field of natural products to catch up with data-centric approaches used in other research fields and further flourish. It is worth pointing out that limiting number of Rosetta hits obtained within this metabolomics dataset is indicative of the potential novel chemistry of the Polar strains which is further supported by the large number of nodes that could not be annotated to specific chemical classes. However, our findings agree with a recent literature review which reported only 29 new metabolites isolated from Antarctic and Arctic bacteria, of which 13 have been discovered from marine actinomycetes [13]. A future direction for NPLinker could be the integration of bioassay data along with metabolomics and genomics datasets, as previously suggested by others [79], which will give the opportunity to users to explore possible MF-GCF links based on bioactivity and target the BGCs and therefore the metabolite(s) responsible for the biological effect. 

## 4. Materials and Methods

### 4.1. Phylogenetic Analysis

Twenty-four rare actinomycete strains and a marine strain of the phylum *Proteobacteria* (Table S1) were previously isolated and taxonomically identified (through 16S rRNA gene sequencing) from the Antarctic and sub-Arctic sediment core collection from two separate studies in conjunction with the Scottish Association for Marine Sciences [11,12]. These strains were selected based on taxonomy and isolation location. Using the 16S rRNA gene sequences, a maximum likelihood (ML) phylogenetic tree was constructed (Kimura 2-parameter model, 1000 bootstraps) using Mega 7 (v 7.0.26) (https://www.megasoftware.net/ (accessed on 9 November 2020)) [80,81] with visualisation and annotation using FigTree (v 1.4.3) (http://tree.bio.ed.ac.uk/software/figtree (accessed on 9 November 2020)). The GenBank accession numbers for the 16S rRNA gene sequences are the following: MT135519 (KRD153), MT135569 (KRD128), MT135795 (KR077), MT135986 (KRD070), MT136106 (KRD026), MT136242 (KRD012), MT136243 (KRD022), MT136510 (KRD096). The remaining strains were deposited in GenBank as mentioned in [11].

### 4.2. Fermentation and Metabolite Extraction

All twenty-five strains were pre-cultured (5 mL, 28 °C, 160 rpm for 7 days) in ISP2 medium [82], ISP3 medium [82] A1M1 medium [27] and 10-fold diluted TSB medium (BD™) prepared in distilled water. Instant ocean (18 g/L) was added in each of them. Each culture [ISP2/ISP3/A1M1/10-fold dil. TSB medium (50 mL) with activated HP-20 resin (Sigma) (2.5 g)] was inoculated (5% *v*/*v* pre-culture) and fermented (14 days, 28 °C, 160 rpm). The culture was then centrifuged (4000 rpm, 20 min), the supernatant removed, and the cell/resin pellet lyophilised until dry (Thermo Savant MicroModulyo, Thermo Fisher Scientific, Waltham, MA, USA). The lyophilised cell/resin pellet was extracted twice with ethyl acetate (Fisher Scientific, Loughborough, UK, reagent grade) (20 mL, 100 rpm, 25 °C). The extracts were combined, dried (under N_2_), the weight recorded and stored (4 °C).

### 4.3. Bioactivity Disc Diffusion Assay

Cultures in TSB (BD™) were prepared for *Escherichia coli*, *Staphylococcus aureus*, *Klebsiella pneumonia*e, *Acinetobacter baumanii,* and *Pseudomonas aeruginosa*, whereas cultures in LB [peptone 10 g/L, yeast extract 5g/L, sodium chloride 5 g/L] (5 mL, 30 °C, 1200 rpm, 12 h) were prepared for *Enterococcus faecalis*. Nutrient agar (NA, 5 mL, ThermoFisher Scientific) was inoculated with 0.1 mg/mL of the pathogen and was poured onto NA Petri plates (10 mL). The ethyl acetate metabolite extracts were re-dissolved in ethyl acetate at a concentration of 5 mg/mL and 20 µL was added onto each sterile disc (5 mm). The plates were incubated overnight at 30 °C and the zones of inhibition were recorded (cm).

### 4.4. Bioactivity Agar Plug Assay

The 25 Polar strains were cultured in ISP2, ISP3, 10-fold diluted TSA and Α1M1 media in 6-well plates (38 mm diameter/3 mL of media) until a uniform lawn was formed (25 °C, 7–14 days). Cultures in TSB (BD™) and LB [peptone 10 g/L, yeast extract 5g/L, NaCl 5 g/L] (5 mL, 30 °C, 1200 rpm, 12 h) were prepared for *Escherichia coli*, *Staphylococcus aureus*, *Klebsiella pneumonia*e, *Acinetobacter baumanii, Pseudomonas aeruginosa* and *Enterococcus faecalis*, respectively. NA (5 mL, Thermo Fisher Scientific) was inoculated with 0.1 mg/mL of the pathogen and poured onto NA (10 mL). Plugs (8 mm) from each bacterial lawn (grown for 14 days) were placed on the seeded pathogen plates and incubated overnight at 30 °C and the zones of inhibition were measured (cm). 

### 4.5. Mass Spectral Data Acquisition

LC–MS/MS was performed using a Thermo Scientific Accela LC system coupled to a Thermo Finnigan LTQ Orbitrap mass spectrometer with an ESI source. Bacterial metabolite extracts and control media extracts (no bacteria) were prepared at 1 mg/mL in ACN and were injected onto an ACE 5 (Hichrom) C18 column (5 µm, 75 × 3.0 mm) using the following gradient: 1–5 min (5% ACN in H_2_O), 5–25 min (5–100% ACN), 25–30 min (100% ACN). Mass data were collected in positive ion mode using ESI and mass range 150–1500 *m*/*z* (15,000 resolution). Data-dependent MS2 scans were obtained using collision-induced dissociation (CID) with an energy of 35 eV and activation time of 30,000 ms for the first, second, and third most intense peaks.

### 4.6. Mass Spectral Data Processing

Mass spectral data were processed using MZmine v2.38 freeware (http://mzmine.sourceforge.net/ (accessed on 9 November 2020)) for peak detection, deconvolution, deisotoping, filtering, alignment and gap filling to make multiple data files comparable. Throughout the data processing, the *m*/*z* tolerance used was 0.01, peaks were detected above 3.00E3 and the minimum time span and tR tolerance was 0.1 min. Mass detection was performed using a centroid mass detector with a noise level set at 2.00 × 10^3^.

### 4.7. Molecular Networking

The MS/MS data were converted from raw to mzXML file format using Proteowizard MSConvert [83] and the data were uploaded to the GNPS server [23]. A molecular network was created with the feature-based molecular networking workflow on the GNPS website (http://gnps.ucsd.edu (accessed on 9 November 2020)). The data were filtered by removing all MS/MS fragment ions within +/−17 Da of the precursor *m*/*z*. MS/MS spectra were window filtered by choosing only the top 6 fragment ions in the +/−150 Da window throughout the spectrum. The precursor ion mass tolerance was set to 0.2 Da and a MS/MS fragment ion tolerance of 0.2 Da. A network was then created where edges were filtered to have a cosine score above 0.6 and at least 1 matched peak. Further, edges between two nodes were kept in the network if and only if each of the nodes appeared in each other’s respective top 10 most similar nodes. Finally, the maximum size of a molecular family was set to 100, and the lowest scoring edges were removed from molecular families until the molecular family size was below this threshold. The spectra in the network were then searched against GNPS’ spectral libraries. The library spectra were filtered in the same manner as the input data. All matches kept between network spectra and library spectra were required to have a score above 0.6 and at least 1 matched peak (https://gnps.ucsd.edu/ProteoSAFe/status.jsp?task=124de327f32f474291a5037f41ac991d (accessed on 9 November 2020)). For molecular network visualisation, Cytoscape version 3.6.1 was utilised [84] where each node corresponds to a consensus spectrum and each edge represents a modified cosine similarity score between nodes. The data used for the molecular networking analysis were deposited in the MassIVE Public GNPS database under access number MSV000086584.

### 4.8. MolNetEnhancer Workflow Description for Chemical Class Annotation of Molecular Networks

To enhance chemical structural information within the molecular network, information from in silico structure annotations from GNPS Library Search and Network Annotation Propagation (NAP) were incorporated into the network using the GNPS MolNetEnhancer workflow (https://ccms-ucsd.github.io/GNPSDocumentation/molnetenhancer/ (accessed on 9 November 2020)) on the GNPS website [31]. Chemical class annotations were performed using the ClassyFire chemical ontology [85]. (https://gnps.ucsd.edu/ProteoSAFe/status.jsp?task=adbadc0707e7449dbe4de1562ecd7bd3 (accessed on 9 November 2020)).

### 4.9. Genomic DNA Extraction

All 25 Polar strains were cultured in ISP2 medium (5 mL, 30 °C, 200 rpm for 3 days). High quality genomic DNA was isolated using an in-house protocol based on chemical cell lysis followed by phenol/chloroform extraction [86]. The *Pseudonocardia* sp. strains underwent cell lysis by vigorous vortexing (10 min) of the bacterial cultures with zirconium oxide beads (~0.5 g) (Sigma-Aldrich Ltd., Dorset, UK). The purity and concentration of the obtained genomic DNA was determined using a Nanodrop 2000 spectrophotometer (Thermo Fisher Scientific) followed by measurements on Qubit 2.0 Fluorometer (Invitrogen, Thermo Fisher Scientific, Waltham, MA, USA).

### 4.10. Genome Sequencing and Alignment 

Whole-genome sequencing was carried out by Microbes NG (https://microbesng.com/ (accessed on 9 November 2020)) as follows: Genomic DNA libraries were prepared using Nextera XT Library Prep Kit (Illumina, San Diego, CA, USA) following the manufacturer’s protocol with the following modifications: two nanograms of DNA instead of one were used as input, and PCR elongation time was increased to 1 min from 30 s. DNA quantification and library preparation were carried out on a Hamilton Microlab STAR automated liquid handling system. Pooled libraries were quantified using the Kapa Biosystems Library Quantification Kit for Illumina on a Roche light cycler 96 qPCR machine. Libraries were sequenced on the Illumina HiSeq using a 250 bp paired-end protocol. Reads were adapter trimmed using Trimmomatic 0.30 with a sliding window quality cut off of Q15 [87]. The closest available reference genome was identified using Kraken [88] the reads were mapped with BWA mem for assessing the quality of the data. De novo assembly of the reads was carried out utilising SPAdes [89]. MeDuSa [90] was utilised for genome scaffolding, using reference strains with >95% similarity based on 16S rRNA sequencing data. The *Pseudonocardia* isolates were not analysed by MeDuSa as no reference strains were identified. The whole genome sequences for the polar strains have been deposited to GenBank with the following accession numbers: SAMN14679891-SAMN14679907 (Table S2).

### 4.11. Biosynthetic Gene Cluster Mining and Comparison

The identification of BGCs was carried out using antiSMASH 5 beta [91]. The variety and number of BGCs each Polar strain was visualised using the Circos diagram [92]. The detected BGCs were grouped into Gene Cluster Families (GCF) using BiG-SCAPE 1.0 beta (Navarro-Munoz et al. 2019), with the underlying assumption that similar BGCs, i.e., BGCs that belong to the same GCF, produce similar metabolites. BiG-SCAPE was run using Longest Common Subcluster alignment mode, and cluster analysis carried out at the default cutoff of 0.3.

### 4.12. Computational Pattern Matching

Computational prioritisation of links between BGCs and candidate products made use of two complementary approaches. Firstly, the standardised strain correlation score described in [42] was used to compute a score between each spectrum and each GCF. The original strain correlation score introduced in [41] is heavily influenced by the number of strains present in each spectrum or GCF making the ranking of links between spectra and a particular GCF problematic. The standardised score overcomes this limitation, permitting a more balanced ranking of spectra for each GCF independent of their size. Significance values for each link were computed as described in [42]. Secondly, a novel approach named Rosetta (code available here: https://github.com/sdrogers/nplinker/tree/master/prototype/rosetta_data_prep (accessed on 9 November 2020)) based upon a set of collated matches between the GNPS [23] library spectra and the MiBIG database of characterised BGCs allows for putative links between individual spectra and BGCs to be highlighted. The set consists of 2960 links, 2069 unique spectra, 249 unique MIBiG IDs. To establish this set of collated links, the structural annotations available for both databases were used. A pair of objects from the two datasets were matched if the first blocks of the InChIKeys of the molecules in the GNPS library spectra and MiBIG validated gene cluster products matched. Matching was restricted to the first block to avoid distinguishing between molecules based on chemical properties that would not show up in the MS/MS spectra (e.g., stereochemistry). With this set of collated links, observed spectra and BGCs were putatively matched as follows: spectral similarity between measured MS2 spectra and the relevant subset of the GNPS spectra was computed using the modified cosine score (equivalent to “Analog search” in the GNPS framework). Results from antiSMASH were parsed to extract the known cluster blast results and Rosetta links between spectra and BGCs were generated where the spectra showed similarity to the GNPS spectrum and the MiBIG entry was found in the known cluster blast record for the BGC. All analysis was performed with the NPLinker framework [42] in which potential can be reported using either one of these two scoring methods, or both simultaneously, with user-defined thresholds.

## Data Availability

The code for Rosetta is available at https://github.com/sdrogers/nplinker/tree/master/prototype/rosetta_data_prep (accessed on 21 January 2021). The genomes have been deposited to GenBank with the following accession numbers: SAMN14679891-SAMN14679907 (Table S2). The GenBank accession numbers for the 16S rRNA gene sequences are the following: MT135519 (KRD153), MT135569 (KRD128), MT135795 (KR077), MT135986 (KRD070), MT136106 (KRD026), MT136242 (KRD012), MT136243 (KRD022), and MT136510 (KRD096) (Figure 1). The LC–MS data are available at the MassIVE dataset under access number MSV000086584.

## Supplementary Materials

The following are available online at https://www.mdpi.com/1660-3397/19/2/103/s1, Figure S1: Molecular network of 3107 parent ions produced by 25 Polar actinomycete strains. Nodes are colour coded based on genus: *Agrococcus*, *Dietzia*, *Halomonas*, *Kocuria*, *Microbacterium*, *Micrococcus*, *Pseudonocardia* and *Rhodococcus*. Grey nodes represent media components, whereas orange nodes represent parent ions that are produced by more than one different medium. Figure S2: Pie chart showing the distribution of parent ions (%) between the 36 chemical class terms shown in the legend as annotated by MolNetEnhancer. The percentage of parent ions with no chemical class match (70.5%) is not shown in the pie chart Each class has been colour coded to match the molecular network generated through MolNetEnhancer workflow analysis (Figure 5B). Table S1: Isolation and collection data of the 25 polar bacteria. Table S2:** Genome quality of the Polar strains (*Pseudonocardia* strains were not analysed by MeDuSa as no reference strains were available). Table S3: Identified BGCs using antiSMASH 5 clusters after genome scaffolding using MeDuSa. Table S4: Bioactive bacterial extracts organised by genus, strain name (KRD) and growth medium ISP3, A1M1, ISP2, and 10-fold dil. TSB. Antibiotic activity against the clinical pathogens *E. faecalis*, *S. aureus*, *K. pneumoniae*, *A. baumannii, P. aeruginosa* and *E. coli* is shown as zones of inhibition (cm) and colour coded by inhibition zone size. Table S5: Putatively identified metabolites using the Rosetta approach.
