# Supplementary material for: Comparative Metabologenomics Analysis of Polar Actinomycetes
Source: Mar Drugs. 2021 Feb 10;19(2):103. doi: 10.3390/md19020103 (PMC7916644; doi:10.3390/md19020103)
Supplement: Supplementary file 1 [file marinedrugs-19-00103-s001.pdf]

Table S1: Isolation and collection data of the 25 polar bacteria.

Ref. No	Closest type stain	Polar region	Location	Year of collection	Length (cm)	Type	Depth (m)	Section (B, M, U)
KRD012	*Micrococcus* sp.	Antarctica	Antarctic PS61-139	N/R	N/R	N/R	N/R	N/A
KRD022	*Micrococcus* sp.	Antarctica	PS67-16A, Agullas Basin	2005	N/R	Box	4730	N/A
KRD026	*Micrococcus* sp.	Antarctica	PS67-16A, Agullas Basin	2005	N/R	Box	4730	N/A
KRD070	*Micrococcus* sp.	Arctic	M5M-75-62-B4, Voring Plateau	2002	N/R	Gravity	1358	N/A
KRD077	*Micrococcus* sp.	Arctic	M5M-75-62-B4, Voring Plateau	2002	N/R	Gravity	1358	N/A
KRD096	*Micrococcus* sp.	Arctic	M5M-127b-99b8, Kongsfjord Outer	2005	N/R	Piston	388	N/A
KRD128	*Micrococcus* sp.	Arctic	M5M-219-143A, Northern Svalbard Margin	2007	N/R	Piston	N/R	N/A
KRD140	*Kocuria* sp.	Antarctica	Antarctic PS66-88A, Weddell Abyssal P.	205	N/R	multi	4932	N/A
KRD153	*Micrococcus* sp.	Arctic	M5M-219-142B	N/R	N/R	N/R	N/R	N/R
KRD162	*Rhodococcus* sp.	Antarctica	PS61 138-8 (BC052-A)	2002	38	Box	4539	B
KRD168	*Pseudonocardia* sp.	Antarctica	PS61 138-8 (BC052-A)	2002	38	Box	4539	B
KRD169	*Pseudonocardia* sp.	Antarctica	PS61 138-8 (BC052-A)	2002	38	Box	4539	B
KRD171	*Halomonas* sp.	Arctic	JR75 (GC067)	2002	77	Gravity	1226	M
KRD172	*Microbacterium* sp.	Arctic	JR75 (GC067)	2002	77	Gravity	1226	M
KRD174	*Microbacterium* sp.	Arctic	JR75 (GC067)	2002	77	Gravity	1226	M
KRD175	*Rhodococcus* sp.	Arctic	JR75 (GC067)	2002	77	Gravity	1226	B
KRD176	*Pseudonocardia* sp.	Antarctica	PS61 134-5 (BC043)	2002	45.5	Box	4060	U
KRD182	*Pseudonocardia* sp.	Antarctica	PS61 134-5 (BC043)	2002	45.5	Box	4060	U
KRD184	*Pseudonocardia* sp.	Antarctica	PS61 134-5 (BC043)	2002	45.5	Box	4060	M
KRD185	*Pseudonocardia* sp.	Antarctica	PS61 134-5 (BC043)	2002	45.5	Box	4060	M
KRD186	*Agrococcus* sp.	Antarctica	PS61 134-5 (BC043)	2002	45.5	Box	4060	B
KRD188	*Pseudonocardia* sp.	Antarctica	PS61 134-5 (BC043)	2002	45.5	Box	4060	B
KRD291	*Pseudonocardia* sp.	Antarctica	PS61 134-5 (BC043)	2002	45.5	Box	4060	B
KRD197	*Rhodococcus* sp.	Antarctica	PS61 140-2 (BC055W)	2002	16	Box	2946	U
KRD202	*Dietzia* sp.	Arctic	JR219 PC141	2010	86.5	Piston	N/R	N/R

N/R: Not recorded, N/A: Not Applicable. Section (B, M, U): section of the core where the sample was taken; B: Bottom, M: Middle, U: Upper

Table S2: Genome quality of the Polar strains. (*Pseudonocardia* strains were not analysed by MeDuSa as no reference strains were available)

Strain	GenBank accession number	Most frequent family (%)	Most frequent species (%)	Number of reads	GC (%)	Number of contigs	Total length (bp)	N50
KRD 012	SAMN14679891	Micrococcaceae (86.32)	*Micrococcus luteus* (86.13)	587934	73,14	54	2428345	152950
KRD 022	SAMN14679892	Micrococcaceae (87.14)	*Micrococcus luteus* (86.94)	991624	72,5	634	2708943	48082
KRD 026	SAMN14679893	Micrococcaceae (84.08)	*Micrococcus luteus* (83.67)	777451	72,72	93	2630609	126029
KRD 070	SAMN14679894	Micrococcaceae (84.26)	*Micrococcus luteus* (84.04)	860196	72,96	61	2506630	120226
KRD 077	SAMN14679895	Micrococcaceae (86.50)	*Micrococcus luteus* (85.18)	897931	72,79	117	2553835	77566
KRD 096	SAMN14679896	Micrococcaceae (87.02)	*Micrococcus luteus* (86.67)	335398	72,99	91	2499004	167875
KRD 128	SAMN14679897	Micrococcaceae (85.88)	*Micrococcus luteus* (85.37)	1120826	73,19	69	2447759	166662
KRD 140	SAMN14679898	Micrococcaceae (34.9)	*Kocuria rhizophila* (32.10)	253418	68,91	36	2743959	503418
KRD 153	SAMN14679899	Micrococcaceae (87.30)	*Micrococcus luteus* (87.09)	2015367	72,87	82	2546919	127028
KRD 162	SAMN14679900	Nocardiaceae (10.26)	*Rhodococcus erythropolis* (3.16)	470260	64,22	110	5901029	339827
KRD 171	SAMN14679901	Halomonadaceae (2.03)	*Halomonas elongata* (1.20)	577377	54,66	57	5542288	425172
KRD 172	SAMN14679902	Microbacteriaceae (7.91)	*Microbacterium testaceum* (4.77)	439847	66,7	38	3362075	3196524
KRD 174	SAMN14679903	Microbacteriaceae (8.27)	*Microbacterium testaceum* (4.93)	1049102	66,53	452	4823384	106614
KRD 175	SAMN14679904	Nocardiaceae (9.63)	*Rhodococcus erythropolis* (2.55)	603595	64,67	82	5449128	432516
KRD 186	SAMN14679905	Microbacteriaceae (6.32)	*Clavibacter michiganensis* (3.34)	293168	70,97	29	3101783	3048703
KRD 197	SAMN14679906	Nocardiaceae (7.72)	*Rhodococcus erythropolis* (1.59)	569376	64,12	296	6781538	736182
KRD 202	SAMN14679907	Nocardiaceae (5.01)	*Rhodococcus pyridinivorans* (0.87)	772984	70,01	101	5564907	341290

N50: the length for which the collection of all contigs of that length or longer covers at least half an assembly

[Type here]

Table S3: Idenitfied BGCs using antiSMASH 5 clusters after genome scaffolding using MeDuSa

Strain	Genus	Total Clusters	NRPS	NRPS-like	Type 1 PKS	Type 2 PKS	Type 3 PKS	hglE-KS	Terpene	Betalactone	Siderophore	Butyrolactone	Arylpolyene	Ectoine	LAP	Lassopeptide	Bacteriocin	Hybrid	Hybrid types
KRD012	Micrococcus	5	0	1	0	0	0	0	1	1	1	0	0	1	0	0	0	0	–
KRD022	Micrococcus	5	0	1	0	0	0	0	1	1	1	0	0	1	0	0	0	0	–
KRD026	Micrococcus	6	0	1	0	0	0	0	1	1	1	0	0	1	0	0	0	1	Arylpolyene-NRPS
KRD070	Micrococcus	5	0	1	0	0	0	0	1	1	1	0	0	1	0	0	0	0	–
KRD077	Micrococcus	5	0	1	0	0	0	0	1	1	1	0	0	1	0	0	0	0	–
KRD096	Micrococcus	5	0	1	0	0	0	0	1	1	1	0	0	1	0	0	0	0	–
KRD128	Micrococcus	5	0	1	0	0	0	0	1	1	1	0	0	1	0	0	0	0	–
KRD140	Kocuria	5	0	1	0	0	1	0	1	1	1	0	0	0	0	0	0	0	–
KRD153	Micrococcus	5	0	1	0	0	0	0	1	1	1	0	0	1	0	0	0	0	–
KRD162	Rhodococcus	21	8	6	1	0	0	0	2	0	0	1	0	1	0	1	0	1	Lantipeptide/Ladderane
KRD171	Halomonas	4	1	0	1	0	0	0	0	1	0	0	0	1	0	0	0	0	–
KRD172	Microbacterium	5	0	2	0	0	0	0	1	0	0	0	0	1	0	0	0	1	t3PKS/ betalactone
KRD174	Microbacterium	5	0	2	0	0	1	0	1	1	0	0	0	0	0	0	0	0	–
KRD175	Rhodococcus	17	6	4	1	0	0	0	2	0	0	1	1	1	0	0	1	0	–
KRD186	Agrococcus	5	0	1	0	0	1	0	1	1	0	0	0	1	0	0	0	0	–
KRD197	Rhodococcus	22	10	2	1	1	0	1	2	0	0	0	0	1	1	0	0	3	Butyrolactone/NRPS, NRPS-like/Arylpolyene, PKS-like/ t1PKS/Arylpolyene
KRD202	Dietzia	7	1	1	1	0	0	0	2	0	1	0	0	1	0	0	0	0	–

Table S4: Bioactive bacterial extracts organised by genus, strain name (KRD) and growth medium ISP3, A1M1, ISP2, and 10-fold dil. TSB. Antibiotic activity against the clinical pathogens *E. faecalis*, *S. aureus*, *K. pneumoniae*, *A. baumannii*, *P. aeruginosa* and *E. coli* is shown as zones of inhibition (cm) and colour coded by inhibition zone size.

Genus	Strain	Growth medium	*E. faecalis*	*S.aureus*	*K. pneumoniae*	*A. baumannii*	*P. aeruginosa*	*E.coli*
Micrococcus sp.	KRD026	ISP2	-	0,5	-	-	-	-
		M1	-	0,4	-	-	-	0,3
Micrococcus sp.	KRD070	ISP2	-	0,3	-	-	-	0,1
Micrococcus sp.	KRD077	ISP2	-	0,3	-	0,1	-	0,1
		ISP3	-	-	-	-	0,2	-
		M1	-	-	-	-	0,3	-
		TSB	-	-	-	-	0,2	-
Micrococcus sp.	KRD096	ISP2	-	-	-	-	-	0,1
Micrococcus sp.	KRD153	ISP2	-	0,3	-	-	-	-
		M1	-	-	-	-	0,2	0,1
		TSB	-	-	-	-	0,1	-
Pseudonocardia sp.	KRD168	ISP3	-	0,7	-	-	-	-
Pseudonocardia sp.	KRD182	ISP3	-	0,8	-	-	-	-
		TSB	-	0,7	-	-	-	-
Pseudonocardia sp.	KRD185	ISP3	0,6	-	-	-	-	-
		M1	-	2,1	0,9	1,5	2,1	0,9
Pseudonocardia sp.	KRD188	ISP3	-	0,3	-	-	-	0,2
Rhodococcus sp.	KRD175	M1	-	-	0,4	-	-	-
		TSB	-	-	0,5	-	-	-
Kocuria sp.	KRD140	ISP2	-	0,6	-	-	-	0,4
		ISP3	-	-	-	-	-	0,3
		M1	-	0,4	-	-	-	0,3

Table S5: Putatively identified metabolites using the Rosetta approach.

Compound	GNPS Spectrum ID	GNPS Library ID	m/z	Metabolomics Spectrum Resolver URL
Antimycin-like	225126	CCMSLIB00000569369	547.3815	https://www.google.com/url?q=https://metabolomics-usi.ucsd.edu/mirror?usi1%3Dmzspec:GNPS:TASK-124de327f32f474291a5037f41ac991d-spectra/specs_ms.mgf:scan:225126%26usi2%3Dmzspec:GNPS:GNPS-LIBRARY:accession:CCMSLIB00000569369%26width%3D10.0%26height%3D6.0%26mz_min%3D%26mz_max%3D%26max_intensity%3D150.0%26grid%3Dtrue%26annotate_peaks%3D%5B%5B547.3823%5D,%5B101.0603,219.0767,245.0553,401.2062,547.2642%5D%5D%26annotate_precision%3D4%26annotation_rotation%3D90%26cosine%3Dstandard%26fragment_mz_tolerance%3D0.2&sa=D&ust=1604063429163000&usg=AOvVaw2P1sMf6uISDwba9JbJ0cB_
Conglobatin-like	200494	CCMSLIB00004710288	521.3294	https://www.google.com/url?q=https://metabolomics-usi.ucsd.edu/mirror?usi1%3Dmzspec:GNPS:TASK-124de327f32f474291a5037f41ac991d-spectra/specs_ms.mgf:scan:200494%26usi2%3Dmzspec:GNPS:GNPS-LIBRARY:accession:CCMSLIB00004710288%26width%3D10.0%26height%3D6.0%26mz_min%3D%26mz_max%3D%26max_intensity%3D150.0%26grid%3Dtrue%26annotate_peaks%3D%5B%5B204.8952,284.0693,521.329%5D,%5B521.2615%5D%5D%26annotate_precision%3D4%26annotation_rotation%3D90%26cosine%3Dstandard%26fragment_mz_tolerance%3D0.2&sa=D&ust=1604063429168000&usg=AOvVaw0ECRKh1MCwlrowwvMXBYK
Ectoine	219769	CCMSLIB00000218169	185.1012	https://www.google.com/url?q=https://metabolomics-usi.ucsd.edu/mirror?usi1%3Dmzspec:GNPS:TASK-124de327f32f474291a5037f41ac991d-spectra/specs_ms.mgf:scan:219769%26usi2%3Dmzspec:GNPS:GNPS-LIBRARY:accession:CCMSLIB00000218169%26width%3D10.0%26height%3D6.0%26mz_min%3D%26mz_max%3D%26max_intensity%3D150.0%26grid%3Dtrue%26annotate_peaks%3D%5B%5B125.0711,143.0816%5D,%5B143.1%5D%5D%26annotate_precision%3D4%26annotation_rotation%3D90%26cosine%3Dstandard%26fragment_mz_tolerance%3D0.2&sa=D&ust=1604063428992000&usg=AOvVaw2f3CK1d6FsQ3zgjeEJNFYV

Chloramphe-nicol	111427	CCMSLIB00004710288	380.2794	https://www.google.com/url?q=https://metabolomics-usi.ucsd.edu/mirror?usi1%3Dmzspec:GNPS:TASK-124de327f32f474291a5037f41ac991d-spectra/specs_ms.mgf:scan:219769%26usi2%3Dmzspec:GNPS:GNPS-LIBRARY:accession:CCMSLIB00000218169%26width%3D10.0%26height%3D6.0%26mz_min%3D%26mz_max%3D%26max_intensity%3D150.0%26grid%3Dtrue%26annotate_peaks%3D%5B%5B125.0711,143.0816%5D,%5B143.1%5D%5D%26annotate_precision%3D4%26annotation_rotation%3D90%26cosine%3Dstandard%26fragment_mz_tolerance%3D0.2&sa=D&ust=1604063428992000&usg=AOvVaw2f3CK1d6FsQ3zgjeEJNFYV

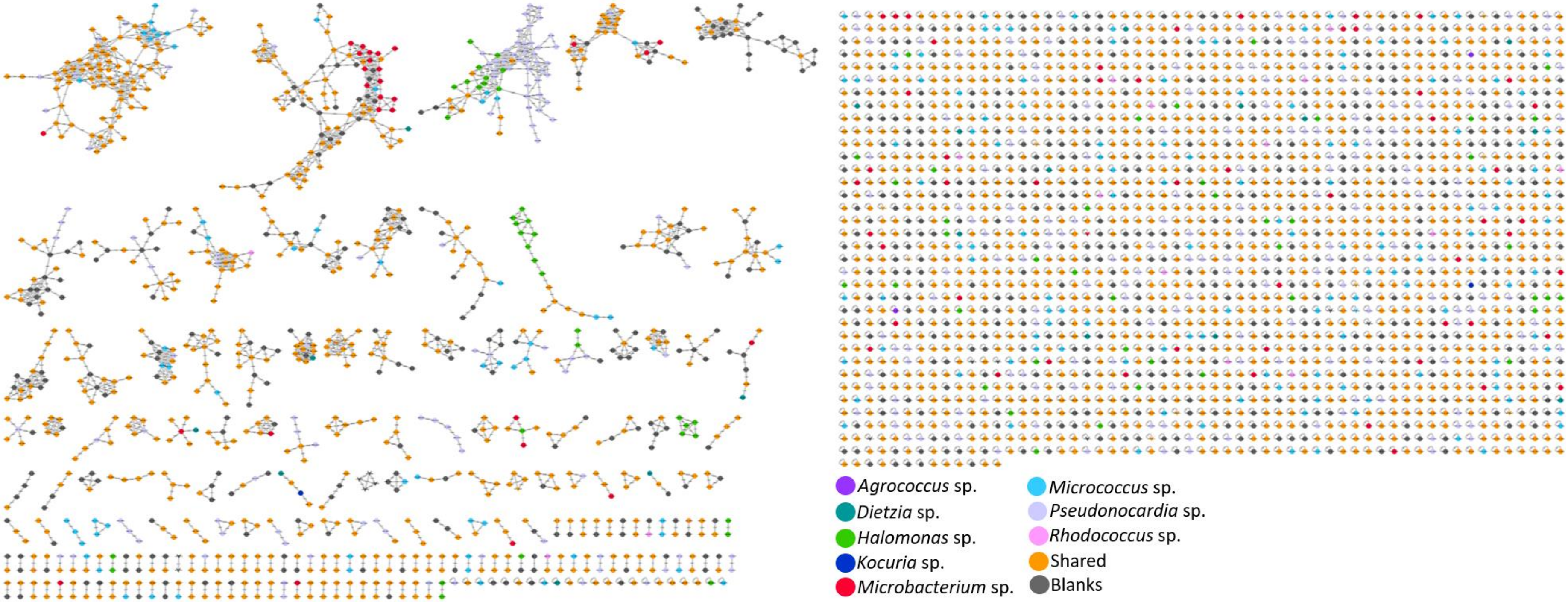

Figure S1: Molecular network of 3107 parent ions produced by 25 Polar actinomycete strains. Nodes are colour-coded based on genus: *Agrococcus*, *Dietzia*, *Halomonas*, *Kocuria*, *Microbacterium*, *Micrococcus*, *Pseudonocardia* and *Rhodococcus*. Grey nodes represent media components whereas orange nodes represent parent ions that are produced by more than one different medium.

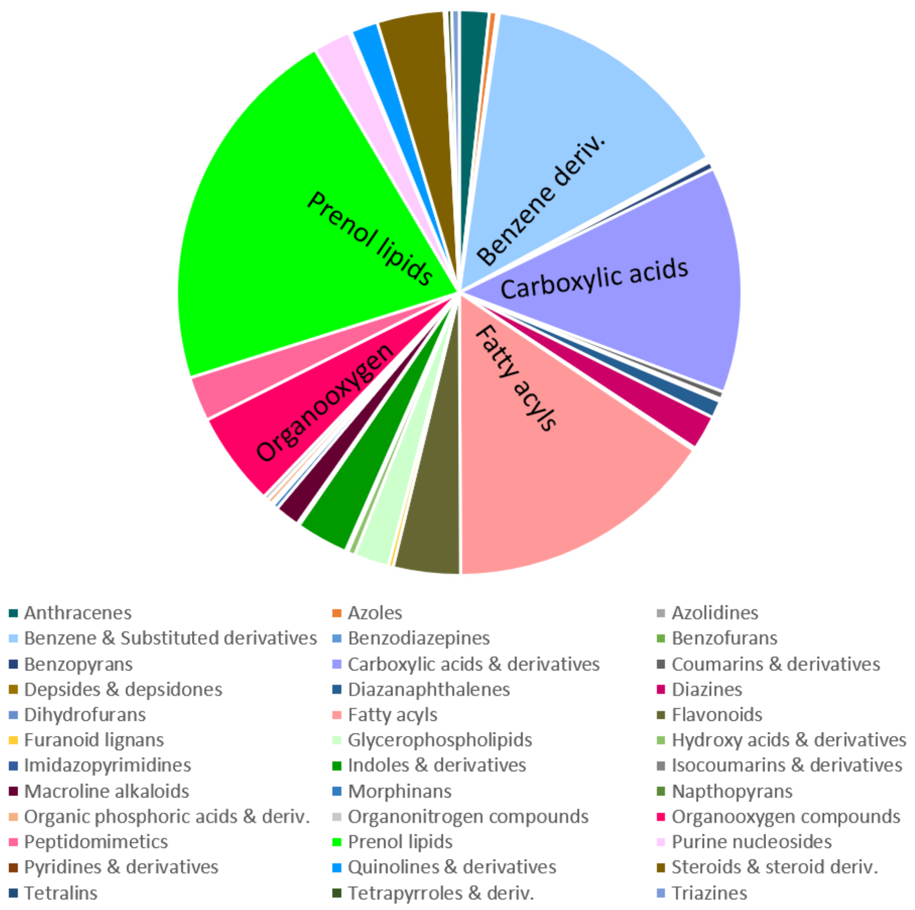

Figure S2: Pie chart showing the distribution of parent ions (%) between the 36 chemical class terms shown in the legend as annotated by MolNetEnhancer. The percentage of parent ions with no chemical class match (70.5%) is not shown in the pie chart. Each class has been colour coded to match the molecular network generated through MolNetEnhancer workflow analysis (Figure 5B)
